# Vascular Homeostasis and Inflammation in Health and Disease—Lessons from Single Cell Technologies

**DOI:** 10.3390/ijms21134688

**Published:** 2020-06-30

**Authors:** Olga Bondareva, Bilal N. Sheikh

**Affiliations:** Helmholtz Institute for Metabolic, Obesity and Vascular Research (HI-MAG) of the Helmholtz Zentrum München at the University of Leipzig and University Hospital Leipzig, Philipp-Rosenthal-Str. 27, 04103 Leipzig, Germany

**Keywords:** vasculature, atherosclerosis, single cell technologies, neurodegeneration, inflammation

## Abstract

The vascular system is critical infrastructure that transports oxygen and nutrients around the body, and dynamically adapts its function to an array of environmental changes. To fulfil the demands of diverse organs, each with unique functions and requirements, the vascular system displays vast regional heterogeneity as well as specialized cell types. Our understanding of the heterogeneity of vascular cells and the molecular mechanisms that regulate their function is beginning to benefit greatly from the rapid development of single cell technologies. Recent studies have started to analyze and map vascular beds in a range of organs in healthy and diseased states at single cell resolution. The current review focuses on recent biological insights on the vascular system garnered from single cell analyses. We cover the themes of vascular heterogeneity, phenotypic plasticity of vascular cells in pathologies such as atherosclerosis and cardiovascular disease, as well as the contribution of defective microvasculature to the development of neurodegenerative disorders such as Alzheimer’s disease. Further adaptation of single cell technologies to study the vascular system will be pivotal in uncovering the mechanisms that drive the array of diseases underpinned by vascular dysfunction.

## 1. Introduction

**Figure 1:**
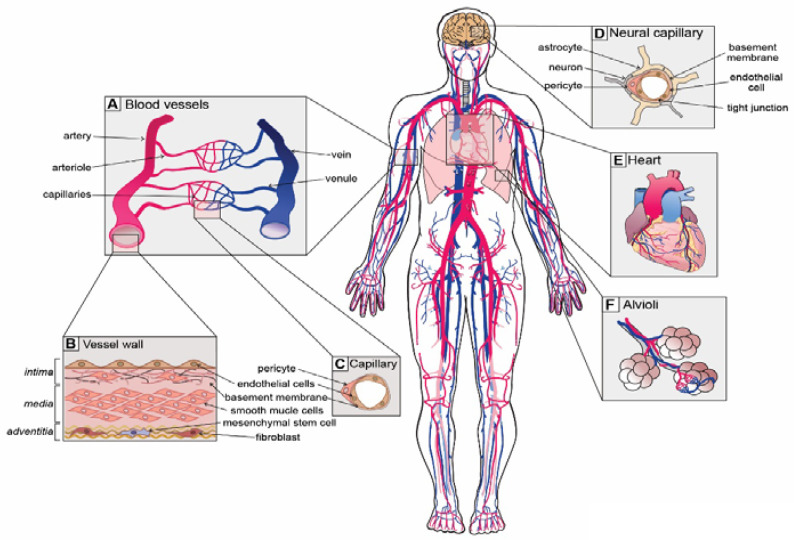
Unique vascular beds in the human body. (**A**) Blood vessels are zonated and display unique cellular phenotypes and functionality. The 5 major zonation states of vessels are arteries, arterioles, capillaries, venules and veins. (**B**) Walls of arterial vessels are typically composed of 3 layers: tunica intima, tunica media and tunica adventitia. The intima is the innermost layer formed by endothelial cells that are in direct contact with the blood. The intima layer is mounted on the basement membrane, which is filled with fibro-elastic extracellular matrix, pericytes and smooth muscle cells. Media, the middle contractile layer, is composed of smooth muscle cells that provide support and flexibility to the vessel. Adventitia, the outmost layer of connective tissue surrounding the vessel, contains fibroblasts, a few mesenchymal stem cells and neurons. (**C**) Capillaries, the smallest blood vessels, are involved in direct solute exchange with the tissue. Capillaries possess a single layer of ECs that is surrounded by basement membrane and contains extracellular matrix and pericytes. Pericytes regulate the permeability of capillaries and their precise density varies from organ to organ. (**D**) Neural capillaries are characterized by an unfenestrated structure and ECs with tight junctions. Neural capillaries are densely populated by pericytes and are often contacted by astrocytes and microglia. (**E**) The heart is the central organ in the cardiovascular system that pumps blood through the whole body, and its function is supported by coronary arteries. (**F**) Lungs possess specialized vasculature that enables oxygen and carbon dioxide exchange between alveoli and pulmonary capillaries.

A healthy and functioning vascular system is critical for ensuring that sufficient nutrients and oxygen reach the ~37 trillion cells in our bodies and the cellular waste products are efficiently removed. To service such a colossal quantity of cells, there is over 100,000 km of vasculature present within our bodies [1], which forms a continuum of vessels from arteries to arterioles, capillaries, venules and veins (Figure 1A) [2]. Differences in vascular cell phenotypes along the artery to vein axis are referred to as vascular zonation and correspond to unique cellular subtypes in each section of the vasculature (Figure 1A) [3]. Blood vessels are generally composed of up to three distinct regions or layers: (i) the tunica intima, a monolayer of flat squamous endothelial cells (ECs) that are in direct contact with the blood stream and mounted on a fibro-elastic basement membrane filled with extracellular matrix; (ii) the tunica media, composed of vascular smooth muscle cells (SMCs) and pericytes; and (iii) the tunica adventitia, composed of connective tissue, including fibroblasts and mesenchymal stem cells (MSCs), as well as lymphatic and neural plexi (Figure 1B,C) [2].

Although the general structure of blood vessels is somewhat conserved throughout the body, each organ has unique functions and demands on the vascular system. For instance, arteries and arterioles, the so-called resistance vessels, endure high pressure and shear stress [4]. The pressure and shear stress gradually reduce towards the veins, which endure up to 70-fold less pressure than arteries [4]. Due to the necessity of withstanding such high forces, arteries and arterioles possess a thick media layer with numerous SMCs that provide elastic support to the vessel walls. Capillaries, on the other hand, are the smallest and thinnest vessels. They only possess an intima layer covered with basement membrane and are supported by pericytes (Figure 1C). Together, SMCs and pericytes constitute the mural cell population that promotes EC differentiation, maintains vascular tone and regulates the permeability of capillaries [5,6]. Consistent with their morphology, capillaries are the major sites of nutrient and gas exchange.

Blood vessels display specializations that are closely associated with organ function. For instance, vessels of the kidney glomerulus need to interact with podocytes and allow filtration of blood and reabsorption of fluids. Therefore, capillary ECs in the kidney are highly fenestrated and permeable [7]. In contrast, the brain has a highly vulnerable environment and is thus afforded protection via the blood–brain barrier (BBB) that restricts the movement of cells, metabolites, infectious agents and proteins in and out of the brain [8,9]. Consistently, ECs of the BBB possess highly specialized non-fenestrated vasculature with specialized tight junctions to maintain the selective nature of the BBB [8,9]. To achieve unique organ-specific functions, vascular cells from distinct organs display unique gene expression programs and phenotypic heterogeneity [10], which will be discussed further in this review.

Given the critical role of blood vessels in maintaining homeostasis, deregulation of vascular function is associated with numerous disorders such as atherosclerosis [11] and associated complications such as ischemia, thrombosis, stroke and myocardial infarction [12], as well as neurodegeneration [13], age-related cognitive decline [14] and cancer [15]. However, due to technical limitations, many of the molecular and subtle phenotypic changes in diseased vascular cells remained unknown. Recent advances in genomics and molecular biology techniques such as single cell sequencing have opened new avenues for researchers, allowing characterization of the precise cellular composition of various tissues, including the detection of rare vascular cell subtypes [3,10]. Importantly, single cell technologies have made it possible to identify cellular heterogeneity, as well as phenotypic and transcriptional changes in the context of healthy and diseased conditions. Through these analyses, researchers have begun to unravel novel molecular mechanisms that underlie the healthy development of the vascular system, as well as changes associated with pathological states.

Single cell technologies now span the analyses of RNA, chromatin accessibility, whole genome DNA sequencing and DNA methylation. In this review, we focus on the biological insights garnered from single cell studies, rather than their technical aspects, which have been recently reviewed elsewhere [16,17,18]. We cover studies collating the diversity of vascular cells through the use of single cell and complementary genomics technologies. We discuss the lessons learnt from these novel data in the context of vascular homeostasis. Given the involvement of vascular dysfunction in a range of diseases, we discuss pathological mechanisms underlying vascular dysfunction and inflammation in atherosclerosis, cardiac and pulmonary disease, as well as neurodegeneration. We highlight recent advances in our understanding of the inflammatory pathways in vascular cells that drive these pathologies and discuss important questions that will be critical towards progressing our understanding of vascular function and disease.

## 2. Overview of Single Cell Analyses

Although numerous methods for single cell analyses have now been established, all methods generally utilize a similar design [16]. Single cells are isolated by tissue dissociation and physically separated into individual wells of a collection plate through fluorescence-activated cell sorting (FACS), or encapsulated in droplets using droplet-based technologies (e.g., inDrop, 10x Genomics). For transcriptome analyses, RNA from each cell is isolated, reverse transcribed and barcoded with unique cell-specific oligonucleotides. Moreover, to overcome the PCR duplication artifacts of the extremely low RNA content of single cells, unique molecular identifiers (UMIs) are introduced into the cDNA. Barcoded cDNA from multiple cells is then mixed and libraries are produced for sequencing. Following sequencing, gene transcripts from each unique cell are identified based on the corresponding barcodes. The identity of cells is determined based on their gene-expression profile as well as the expression of known cell markers. The transcriptional profile of each cell type can then be compared between different conditions, for example between diseased and healthy states.

There are unique benefits of single cell approaches over traditional “bulk” analyses that investigate whole tissues or FACS-enriched cell populations [19]. Single cell analyses deconvolute the full complexity of tissues by resolving and characterizing rare cell populations, which would be otherwise masked by more abundant cell types when undertaking analyses of whole complex tissues. Furthermore, through established bioinformatic pipelines, single cell analyses can pick up subtle phenotypic shifts in diseased conditions as well as intermediate cell states during disease onset or during normal cellular differentiation [20]. This is particularly important when studying the molecular mechanisms that induce the transition from a healthy to a diseased cell state. Thus, while “bulk” analyses remain a powerful tool for understanding the average molecular changes in a tissue, single cell analyses allow the identification of cellular heterogeneity as well as phenotypic and transcriptional transitions in individual cell types.

## 3. Single Cell Technology towards Creation of a Comprehensive Vascular Cell Atlas

The first large-scale initiative to produce a reference single cell atlas of healthy wild type mice was pioneered by the Tabula Muris Consortium that sequenced more than 100,000 cells from 20 mouse organs [21]. An analogous Human Cell Atlas consortium [22,23], together with other datasets developed by independent laboratories [24,25,26,27,28], is already on the way to producing a comprehensive single cell atlas of all human tissues. While these single cell RNA (scRNA)-seq databases provide an extremely valuable resource, the vascular system remained to be covered in depth, as vascular cells represent only a minor proportion of an organ. Vascular cells are generally grouped together in global databases and consequently their heterogeneity is overlooked, as bioinformatic algorithms typically do not distinguish between the vascular subtypes amongst the huge diversity of other, more common cell types in whole organs. Thus, considerable effort has since been spent on generating a vascular cell atlas.

Kalucka and colleagues focused on characterizing EC heterogeneity by analyzing more than 32,000 ECs from 11 mouse tissues including the brain, spleen, testis, lung, kidney and heart [10]. They uncovered that EC gene expression profiles are highly tissue dependent. Nevertheless, there were strong similarities in ECs isolated from the small intestine and colon, skeletal muscle and heart, as well as the brain and testis. Moreover, the authors detected specialized EC subtypes in the intestine (*Aqp7^+^*, *Madcam1^+^*) and brain (*Plvap*-high, *Esm1*-high), as well as proliferative and activated EC populations in various organs. Although this is a valuable resource for the field, the heterogeneity of vascular cell types such as pericytes, SMCs and peri-vascular fibroblasts remains to be systematically compared between different organs using similar techniques.

Considerable attention has been paid to the brain due to its highly specialized vasculature that is critical for maintaining a homeostatic neural environment. Vanlandewijck and colleagues isolated ECs (*Cldn^+^* cells), SMCs (*Tagln^+^* cells), pericytes (*Pdgfrb^+^*, *Cspg4^+^ cells*) and fibroblasts (*Pdgfra^+^* cells) from the adult brain by FACS and analyzed them via scRNA-seq [3]. By coupling their transcriptomics data with known EC and mural cell markers, they were able to expand vascular zonation markers using bioinformatic algorithms (Table 1). In addition, these analyses also uncovered zonation-specific transcription factors and transporters enriched in neural ECs and mural cells.

In addition to global EC databases, vascular cells in the adult human heart have been recently annotated [30]. Comprehensive single cell and single nuclei analyses of six distinct regions of the heart uncovered heterogeneity in all major cell types depending on their spatial location and function [30]. In addition to the expected differences between atrial and ventricular cardiomyocytes [31], vascular cells, which constitute approximately 7% of cells in this dataset, displayed zonation-based clustering with the highest proportion of ECs identified as capillary ECs (53%) [30]. Moreover, ECs, SMCs and pericytes exhibited chamber specificity, showing distinct gene expression profiles between atrial and ventricular counterparts. This study emphasizes the importance of single cell approaches to study vascular cells due to their heterogeneity and tissue-specific function.

Single cell technologies have started to uncover critical insights into numerous aspects of vascular function and homeostasis in healthy organisms. In addition, studies are now utilizing single technologies to interrogate phenotypic changes in vascular cells in diseased states. In the sections below, we focus specifically on the themes of chronic vascular dysfunction, which promotes and accompanies atherosclerosis, myocardial infarction and cognitive disorders such as Alzheimer’s disease.

## 4. Vascular Inflammation

The vascular system not only supplies our bodies with nutrients and oxygen, but is also directly involved in the execution of immune reactions to pro-inflammatory stimuli. There are a multitude of stimuli that lead to vascular inflammation including alterations in blood flow and shear stress, increased concentration of low-density lipoproteins (LDLs), metabolic changes such as increased fatty acids, hypertension, agiotensin II signaling, as well as pro-inflammatory cytokines and chemokines that are upregulated in response to viral or bacterial infections [11,32,33,34]. Vascular inflammation is accompanied by active attraction of circulating leukocytes to the site of injury and their transmigration into the intima of the vessel wall, to clear the tissue from the source of inflammation and dead cells and ultimately resolve the inflammation. However, if the inflammation transitions to a chronic state, it can lead to adverse outcomes through the development of vascular diseases like atherosclerosis. Here, we will discuss the role of vascular inflammation in atherosclerosis, myocardial infarction, pulmonary arterial hypertension and neurodegeneration, and focus on new insights obtained from single cell studies.

### 4.1. Atherosclerosis

Atherosclerosis is a common, multi-factorial disorder that typically underlies ischemic heart disease, stroke and peripheral artery disease. Atherosclerosis affects arteries and arterioles by narrowing and stiffening of the vessels due to the build-up of lipid-rich plaques. According to the well-established dogma, atherosclerosis starts with EC dysfunction in response to increased levels of oxidized low-density lipoproteins (LDLs) in the blood stream [35]. ECs sense and uptake toxic LDLs, which promote oxidative stress, a reduction in nitric oxide synthesis and increased apoptosis. LDL-loaded ECs upregulate adhesion molecules like ICAM1, VCAM1 and E- and P-selectins, as well as pro-inflammatory cytokines such as CCL2 (MCP1) and IL1β, and the complement system. These changes in EC phenotype promote attraction, attachment and slow rolling of circulating immune cells on the vessel wall [36]. An inflammatory state leads to opening of adherent junctions, promoting immune cell transmigration into intima of the vessel wall, where monocytes differentiate into macrophages and thus facilitate an inflammatory state and clearance of excessive lipids and dead cells [37]. Macrophages that uptake LDLs transform into foam cells and are typical of atherosclerotic plaques [37].

**Figure 2:**
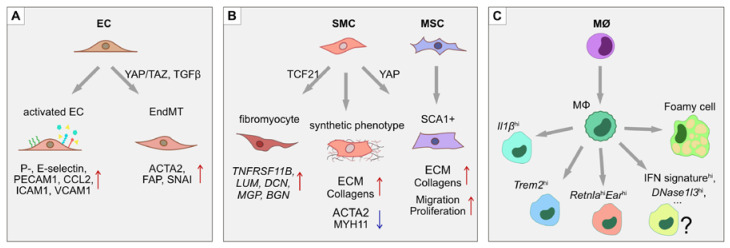
Phenotypic switches during atherosclerosis. In atherosclerotic lesions, several processes of phenotypic modulation and trans-differentiation take place. (**A**) ECs upregulate adhesion molecules such as ICAM1, VCAM1, E- and P-selectins, as well as secreting pro-inflammatory cytokines CCL2 and IL1β, which help attract leukocytes. In atherosclerotic plaques, ECs also undergo endothelial-to-mesenchymal transition (EndMT) through the activation of YAP/TAZ- and TGFβ-driven pathways. The EndMT transitions are characterized by the loss of endothelial identity markers such as PECAM1, together with the upregulation of mesenchymal markers α-smooth muscle actin (ACTA2), fibroblast activation protein (FAP) and the SNAI transcription factors. (**B**) SMCs undergo a phenotypic switch from a contractile to a synthetic state by increasing production of ECM proteins and downregulating *MYH11*. Moreover, a subset of SMCs in the atherosclerotic lesion express the stem cell marker SCA1, suggesting either mesenchymal stem cell differentiation into SMCs, or de-differentiation of SMCs towards an MSC-like state. (**C**) Monocytes, upon transmigration into the intima of the lesion, differentiate into macrophages that display at least 3 unique subsets: (i) resident-like anti-inflammatory cells, (ii) pro-inflammatory *Il1β*^hi^ cells, and (iii) *Trem2*^hi^ cells. Macrophages that take up low-density lipoproteins (LDLs) upregulate lipid metabolism related genes and take on a “foamy macrophage” phenotype.

SMCs, on the other hand, contribute to vascular wall remodeling. SMCs migrate into the intima of the lesion area, where they undergo a phenotypic switch and start to excessively produce extracellular matrix (ECM) and proliferate. Some SMCs reportedly express macrophage markers in response to LDLs and pro-inflammatory stimuli, suggesting the possibility of trans-differentiation [38,39]. The opposite also seems to take place, where macrophages and myeloid cells begin to express a number of SMC markers [40,41,42]. Moreover, ECs have been shown to undergo endothelial-to-mesenchymal transition (EndMT) in atherosclerotic plaques in vivo, as well as in response to disturbed flow in vitro via activation of Yes-associated protein/transcriptional coactivator with PDZ-binding motif (YAP/TAZ) complex and TGF-β signaling [43,44]. ECs acquire a contractile phenotype with expression of SMC-specific genes like α-smooth muscle actin (ACTA2), connective tissue growth factor (CTGF) and fibroblast activation protein (FAP), while losing EC-specific gene expression (Figure 2) [43]. Mesenchymal stem cells (MSCs) normally reside in the adventitia of the vessel and are also found in atherosclerotic lesions, but it is unclear if they migrate from the adventitia or originate from trans-differentiation of ECs or SMCs [45,46]. Further plaque growth is accompanied by lipid accumulation, calcification, apoptosis and necrotic core formation. Infiltrating immune cells secrete metalloproteinases that digest and thin the fibrous cap, thus promoting plaque rupture. If the plaque is ruptured, it leads to thrombus formation and ultimately vessel occlusion that can result in adverse outcomes such as stroke and myocardial infarction.

As it becomes clearer that atherosclerosis involves cellular plasticity and phenotypic transitions including EndMT, SMCs and immune cell trans-differentiation, single cell technologies are becoming increasingly instrumental in addressing heterogeneity of atherosclerotic plaques. A number of studies have begun to utilize scRNA-seq to investigate total diseased arterial wall tissue [47], samples enriched for SMCs [48,49], and purified cell populations of infiltrating immune cells [50,51] to gain insights into the pathophysiological mechanisms underlying atherosclerosis.

To understand atherosclerosis development in controlled animal models, Kalluri and co-workers performed a single cell analysis of more than 10,000 cells derived from aortas of 12-week-old wild type C57/BL6 mice on either chow or Western diets [47]. They detected all major cell types in the plaque including ECs, SMCs, fibroblasts, monocytes and macrophages. Moreover, they discovered three functionally distinct EC populations: (i) *Vcam1*-enriched population, (ii) EC population with higher lipid metabolism and angiogenesis related genes and (iii) a lymphatic population. In comparison to mice on the control diet, Western diet-fed mice developed changes in EC expression with increased transcription of contractile genes that might be related to EndMT.

Other studies have focused on the non-EC compartments in the atherosclerotic plaques. Working on the *ApoE*^−/−^ atherosclerosis mouse model, Gu and co-workers found that non-immune cells also appear in a pro-inflammatory and stem-/progenitor-like state that is typified by the expression of stem cell antigen 1 (SCA1) in SMCs [52]. Moreover, isolated SCA1^+^ SMCs had high CCL2 expression in vitro, consistent with the pro-inflammatory signature. Other cell types such as resident macrophages were also in an activated pro-inflammatory state [52]. While Western diet or *ApoE* knockout mouse models do not result in formation of progressive atherosclerotic lesions, these models nevertheless provide important insights into its early development and first-wave responses of ECs and SMCs to atherosclerosis development.

#### 4.1.1. Smooth Muscle Cells in Atherosclerosis

One important question in the field has been the origin of SMCs found in atherosclerotic plaques. In order to answer this question, several studies have combined scRNA-seq with *Myh11*-driven SMC lineage tracing in *ApoE*^−/−^ animals [48,49]. Firstly, SMCs from the aortic arch and descending thoracic aorta show transcriptional differences suggesting the presence of SMC subtypes. Secondly, a rare subset of progenitor-like SCA1^+^ cells is present in plaques, which is characterized by low contractile function and increased expression of genes related to migration, proliferation and ECM production [48]. SCA1^+^ SMCs are also increased in atherosclerotic lesions of *ApoE*^−/−^ mice, suggesting that regulation of *Sca1* expression may contribute to the switch from contractile to the activated SMC phenotype. Consistently, SCA1^+^ SMCs are activated upon arterial injury and are crucial for vascular repair and regeneration mediated by YAP1, a member of the Hippo pathway [53]. Together, the presence of SCA1^+^ cells in atherosclerotic lesions might be viewed as an exaggerated repair response to chronic inflammation and injury.

Recently, attention has shifted towards identifying important regulatory molecules via scRNA-seq analysis. Transcription factor 21 (TCF21) is highly expressed within atherosclerosis lesions and coincides with SMC markers [54]. A recent scRNA-seq study found TCF21 to mediate the SMC phenotypic switch and fibrous cap formation [49]. The authors discovered a phenotypic switch of SMCs in atherosclerotic plaques towards a fibroblast-like transcriptional signature that they named “fibromyocyte”. Fibromyocytes were characterized by upregulation of fibroblast marker genes lumican (*Lum*), decorin (*Dcn*) and biglycan (*Bgn*) and increased expression of *Tcf21*. On the other hand, SMCs did not shift towards a macrophage phenotype. Despite expressing the *Lgals3* macrophage marker, SMCs did not acquire expression of other macrophage markers including *Cd68*, *Cd16*, *Cd32*, *Cd11b*, *Cd64* and *Cd86*. Thus, these results seemingly oppose the hypothesis of SMC-to-macrophage trans-differentiation [38]. The existence of “fibromyocytes” was further confirmed in four human atherosclerotic plaque samples by scRNA-seq and complimentary *in situ* RNA hybridization, where osteoprotegerin (*TNFRSF11B*) was found to mark the fibromyocyte cell population [49]. Moreover, SMC-specific *Tcf21* knockout in mice led to a reduced fibromyocyte population at the fibrous cap and in the atherosclerotic lesion, while overexpression of *TCF21* in the human coronary artery SMC cell line led to the upregulation of *LUM*, *DCN* and *MGP* fibromyocyte genes, suggesting a conserved role of TCF21 in mouse and human models [49]. Consistent with findings in atherosclerosis, the involvement of TCF21 in fibrosis has been reported in a number of other organs [55,56,57]. Together, these studies suggest that TCF21 is an important player in atherosclerosis-related fibrosis and might modulate plaque stability via fibrous cup support.

Taken together, SMCs found in atherosclerotic plaques undergo a phenotypic switch and acquire stem cell-like and/or fibroblast-like markers. However, future work is still required to determine the origin of these cells.

#### 4.1.2. Immune Cells in Atherosclerosis

As atherosclerosis is a chronic inflammatory disease, there is a strong contribution from the immune cell compartment. To characterize the heterogeneity of immune cell infiltrates in atherosclerotic lesions, studies have undertaken FACS isolation of CD45^+^ hematopoietic cells followed by scRNA-seq [50,51]. These analyses have established that atherosclerotic plaques are rich in various immune cell types, including monocytes, macrophages, B-cells, T-cells, NK-cells and granulocytes [50,51]. In turn, each infiltrating immune cell population also displays significant heterogeneity. For instance, three distinct populations of macrophages in atherosclerotic plaques have been described [50]. Of these, two macrophage cell populations were specific to atherosclerotic lesions in *Ldlr*^−/−^ mice maintained on a high fat diet (HFD), namely (i) inflammatory macrophages with high expression of *Il1b* and (ii) macrophages with high expression of triggered receptor expressed on myeloid cells 2 (*Trem2*^hi^). Importantly, these two macrophage populations were also independently detected in the *ApoE*^−/−^ high fat diet atherosclerosis model [50], strengthening the claim that these populations may be a general characteristic of atherosclerotic plaques. Furthermore, increased numbers of foamy macrophages are also found in plaques and their number correlates with the size of the atherosclerotic lesion [51]. Consistent with their LDL-loaded composition, foamy macrophages display increased expression of fatty acid and cholesterol transport and uptake genes. In contrast, inflammation-related genes including *Il1b* are most highly expressed in the non-foamy macrophage populations [51].

Due to high numbers of immune cells in the atherosclerotic lesion, it is debatable if all immune cells originate from the blood or whether they might be derived from resident macrophages and proliferate in the lesion itself [58]. To address this question, Lin and colleagues examined the fate of infiltrating CX3CR1^+^ CD11b^+^ monocytes and macrophages during atherosclerosis progression [59]. Ten different subpopulations of myeloid lineage cells were detected by dataset clustering [59], including the three previously reported macrophage populations (resident-like, inflammatory, and *Trem2*^hi^) [50]. Novel macrophage populations including *DNase1l3*^high^, IFN^high^, as well as *Retnla*^hi^/*Ear*^hi^, were strongly enriched in progressive plaques compared to regressive ones [59]. Interestingly, a highly proliferative “stem-like macrophage” population was detected in both progressing and regressing plaques [59], suggesting that macrophage precursors can proliferate within atherosclerotic plaques and give rise to distinct types of macrophages.

Taken together, scRNA-seq studies of atherosclerotic plaques provide new evidence of complex and intricate regulation of cell phenotypes and function in response to chronic inflammation (Figure 2). Despite the important insights gained from scRNA-seq studies on atherosclerosis models, there remain a number of outstanding questions. For instance, while the final diseased state in atherosclerotic animals has been well defined, the molecular changes leading to the development of the atherosclerotic plaque remain to be investigated in detail. It would be highly informative to produce a comprehensive scRNA-seq based timeline of atherosclerotic lesion development to uncover the precise sequence of molecular events that drive changes in cellular phenotypes. Moreover, it is of paramount importance to study how the vast number of cell types, each with unique disease-specific phenotypes, communicate with each other. These intercellular communication networks remain to be mapped. Further, there are only limited data available in human subjects. While the mouse models provide significant molecular insights, future studies utilizing primary human plaques, together with single cell RNA and chromatin analyses, will reveal whether molecular networks discovered in animal models also hold true in human samples.

### 4.2. Cardiac Vasculature in Disease

As a disease that impairs blood circulation, atherosclerosis has direct effects on the fitness of the cardiovascular system, and on the heart in particular. Vessel wall thickening and stiffening eventually results in increased blood pressure and potentially heart failure. Unstable atherosclerotic plaques may rupture and thereby increase the risk of thrombosis, acute coronary syndrome and myocardial infarction (MI) due to insufficient blood supply through coronary arteries [60]. MI leads to muscle damage and scarring, as well as the loss of contractile and conductive function of the heart, ultimately resulting in cardiac insufficiency and the need for heart support or replacement. As any scar tissue, cardiac scars after MI are characterized by increased migration and proliferation of fibroblasts, extensive ECM production and inflammation targeted to remove dead cells [61].

Changes in cell composition and phenotypic switches after MI have been addressed by a number of studies, where an influx of immune cells [62] and increased fibrosis [63,64] in the scar have been reported. Scar formation is propagated via fibroblast activation modulated by periostin (*Postn*) and TGFβ-WNT signaling pathways, as well as fibroblast trans-differentiation into myofibroblasts, which is accompanied by the acquisition of SMC markers [65,66]. There is also new evidence of an increase in the EC population starting as early as day 5 post-MI [64]. This change can be related to re-vascularization of the scar tissue, which is critical for the healing process. Indeed, increased EC proliferation and clonal expansion of EC into the border zone of MI was reported seven days post-MI in animal models [67]. ScRNA-seq analysis of FACS-sorted ECs from healthy and ischemic hearts at seven days post-MI detected 10 distinct EC subpopulations, including a small CD45^+^ population [67], which could represent either a phenotypic switch of EC into an inflammatory state, or infiltrating immune cells taking on some EC-like characteristics. Other EC subpopulations include proliferative, angiogenic and regenerative signatures, which represent a neovascularization response to injury. Moreover, ECs with high expression of cardiomyocyte genes were also detected, suggesting a mechanism to compensate for myocyte loss. These ECs display strong expression of inflammatory mediators to attract immune cells and facilitate clearance of debris in the scar tissue.

Taken together, cardiac injury is characterized by dynamic changes in the EC population and multiple events of trans-differentiation that are mostly related to fibroblasts. However, new data on scar neovascularization promise novel opportunities for post-MI treatment and recovery, emphasizing the importance of vascular cells in the period following MI.

### 4.3. Pulmonary Arterial Hypertension

Pulmonary vasculature undertakes the essential function of oxygen and carbon dioxide exchange between the blood and air at the interface of alveoli and alveolar capillaries. However, a number of factors, including genetics, side effects of some drugs and pre-existing conditions such as heart or lung disease, can lead to the development of pulmonary arterial hypertension (PAH) [68]. PAH is characterized by a chronic increase in pulmonary artery pressure and vascular resistance despite normal heart function. It is accompanied by oxidative stress and vascular inflammation affecting all three layers of the vascular wall, which leads to irreversible vascular remodeling and obliteration of pulmonary arterioles, and ultimately to lung and left ventricle dysfunction [69]. Consistently, PAH development is associated with vasoconstriction, increased ECM production, EndMT, upregulation of inflammatory modulators, immune cell infiltration and extensive EC, SMC and pericyte migration and proliferation that is mediated by BMP-TGFβ signaling, as well as growth factors such as PDGF and FGF [70].

Due to recent efforts in assembling single cell lung atlases, the precise tissue composition of the lungs in humans, mice, rats and pigs has been described [71,72,73]. More recent studies have also begun to focus on diseased states such as PAH. ScRNA-Seq analyses in human PAH patients have uncovered the upregulation of angiogenesis-associated genes in ECs such as endoglin (*ENG*), *NOTCH1* and *KDR*, together with genes involved in SMC migration and proliferation such as *PDGFB* and *FGFR1*, as well as vasoconstrictor endothelin 1 (*EDN1*) [74]. Interestingly, apelin (*APLN*) and apelin cleaving enzyme lysosomal pro-X carboxypeptidase (*PRCP*) were elevated in PAH samples [74], contradicting the previously reported reduction in *APLN* and *PRCP* expression in pulmonary artery ECs isolated from PAH patients in vitro and the regression of PAH in mice after APLN administration [75,76]. Genes upregulated in SMCs and pericytes were related to ECM components that support vessel wall remodeling and increased ECM stiffness [74]. Furthermore, bioinformatic analyses of regulatory networks derived a number of transcription factors such as *SOX18*, *STRA13*, *LYL1* and *TFDP1* [74], which are likely to play an important role in ECs in PAH.

Given the cellular complexity of the lungs, a number of scRNA-seq studies have also focused on intercellular communication [71,72,73,77], which is of great importance for understanding the contribution of individual cell types to complex diseases. For instance, the interaction between alveolar type I epithelial cells and capillary ECs through VEGF and semaphorin signaling is strongly conserved across species [77], suggesting the importance of interactions between these two cell types. Additionally, bioinformatic analyses have confirmed that lung ECs are able to detect and process major circulating hormones [73]; Lung ECs express the angiotensin-converting enzyme (ACE) and endothelin-converting enzyme (ECE1), which are utilized for the production of biologically active peptides that can in turn be sensed by pericytes. Furthermore, acetylcholine receptors are expressed by lung ECs, which stimulate the production of the vasodilatory nitric oxide and prostacyclin upon activation [73].

In addition to transcriptional changes, there is growing evidence that epigenetics may also play an important role in ECs during PAH. The histone acetylation mark, histone H3 lysine 27 acetylation (H3K27ac), normally found on active regulatory elements such as enhancers and promoters, has been recently reported to differ in small pulmonary artery ECs isolated from healthy and PAH patients [78]. Intriguingly, genes associated with differentially acetylated enhancers in PAH versus control samples were related to cell proliferation, migration and angiogenesis processes [78]. Moreover, a number of transcription factors including members of the AP1 complex, STAT1, STAT4, RFX3, RFX4, GATA4 and GATA6 showed increased activity in PAH samples based on H3K27ac signal, whereas the VEGF target *NOS3*, the serotonin receptor *HTR1BRC*, and *YAP1* enhancers showed a reduction in H3K27ac signal [78]. Interestingly, the mechanosensitive YAP/TAZ complex responds to increased ECM stiffness in PAH by promoting glycolysis and reducing mitochondrial oxidative phosphorylation in pulmonary arterial ECs and SMCs [79]. Together, these data suggest the involvement of AP1, TGFβ, YAP/TAZ, serotonin and VEGF signaling in PAH, resulting in inflammation, EndMT, vascular stiffness and aberrant angiogenesis. To support the notion of epigenetic modulation in PAH, changes in the expression of chromatin regulators *FAM60A* and *HDAC7* in ECs derived from PAH patients have also been reported [74]. The transcriptional repressor complex member FAM60A responds to hypoxia via recruitment of HDAC7 to the *HIF2A* promoter [80,81], whereas the histone deacetylases HDAC6 and HDAC7 play an important role in regulating sprouting, angiogenesis and endothelial barrier function [82,83,84]. Thus, further investigation of epigenetic mechanisms in EC subtypes through single cell technologies (discussed below) would be important for a better understanding of molecular events underlying the development of PAH.

### 4.4. Vascular Dysfunction in Neural Disorders

Compared to the blood vessels of other organs, the vasculature in the nervous system is unique and underpinned by the blood–brain barrier (BBB). In particular, brain ECs are deficient in fenestrations and possess tight junctions that strongly limit the transport of macromolecules, metabolites and toxins into the brain parenchyma [85,86]. In addition to specialized ECs, neural vasculature possesses particularly high numbers of pericytes, with estimates ranging from one pericyte to every one to three ECs [5]. In comparison, the vessels of skeletal muscle, for example, are thought to have only around one pericyte per 100 ECs. Pericytes are critical for the formation of the BBB during development [87,88,89], and their ongoing functionality is critical for modulating its permeability [90,91]. In addition to the two main vascular cell types, numerous brain cells including astrocytes and microglia have close contacts with the blood vessels and are able to directly modulate their activity (Figure 1D). Consistent with this, changes in the neural metabolic environment [34], neural activity [92], inflammatory signals [93], buildup of toxic products such as β-amyloid [94], and infection [95], can all modulate vascular properties such as blood flow and permeability.

Transcriptional analyses [3,19], together with imaging techniques such as light-sheet microscopy [96,97], are starting to shed important light on the makeup of brain vasculature. Single cell transcriptomics focusing on the mouse brain have revealed that neural EC subtypes exist in a zonated manner [3]. Indeed, there is a clear transcriptomic and phenotypic distinction between arterial ECs, capillary ECs and venous ECs, with 1798 transcripts showing differential expression amongst the major neural EC subtypes. For instance, *Bmx*, *Efnb2*, *Vegfc* and *Sema3g* are highly enriched in arterial ECs, *Mfsd2a* and *Trfc* in capillary ECs, whereas *Nr2f2* and *Slc38a5* are enriched in venous ECs [3]. While there are numerous genes reportedly expressed in a zonated fashion in neural ECs, the precise function of the majority of identified genes in the specification or functionality of EC subtypes remains to be ascertained. In contrast to ECs, mural cells do not show zonation, but rather display two major clusters based on their transcriptional profiles [3]. The major mural cluster consists of pericytes and SMCs associated with capillaries and venous vessels that display high levels of *Pdgfrb*, *Vtn* and *Kcnj8* mRNA levels. In contrast, arteriole and arterial smooth muscle cells make up the second mural cell cluster and express high levels of *Myh11*, *Acta2* and *Myl9* [3]. These data are consistent with unique recruitment mechanisms of SMCs and pericytes to different parts of the zonated vasculature. For instance, depletion of *Pdgfrb* primarily results in the loss of pericytes from capillaries, while mural cell recruitment to arterioles is less affected [89]. While the neural vasculature scRNA-seq dataset provides great insights into neural vascular cells at steady states, precisely which subtype of vascular cells change in pathological conditions and through which molecular mechanisms remains to be identified. This is particularly important given the range of neural pathologies impacted by defective vascular function.

The importance of the neural vasculature in a range of neurological disorders, including vascular dementia [98], Alzheimer’s disease [99,100,101,102], Parkinson’s disease [103], Huntington’s disease [104], amyotrophic lateral sclerosis [105] and age-related cognitive decline [90,100], is starting to become clear. Amongst these disorders, the role of vascular dysfunction in Alzheimer’s disease (AD) has been widely studied. Defects in neural vasculature, including increased permeability are thought to be amongst the earliest changes observed and are thought to precede the occurrence of β-amyloid fibrils [100,102]. Indeed, reduced pericyte functionality in mouse models of AD accelerates AD development [99], while higher levels of pericyte stress, evident through increased CSF concentrations of PDGFRβ, strongly predicts accelerated cognitive decline in humans in the following 4.5 years [102].

While a number of scRNA-seq databases have been developed for human AD [106,107], vascular cells only represent a miniscule proportion of cells analyzed and the underlying changes in neural vascular cells remain to be identified. Nevertheless, there is some evidence from scRNA-seq analyses of brain ECs from aging mouse models that capillary ECs maybe the most vulnerable to age-related decline in vascular function [108]. However, given the recent recognition of the key role that pericytes play in the development of neurodegenerative disorders [102,109], it would be of paramount importance to map pericyte transition from a healthy to a diseased state through single cell analyses.

Although there is now increasing appreciation that vascular defects are key parts of the pathology of neurodegenerative disorders, whether environmental factors could also contribute to neurovascular defects remains unclear. Recent work has suggested that a defective metabolic environment could induce vascular inflammation and increased vascular permeability in the developing brain [34]. Through scRNA-seq and transcriptomic analyses of FACS-isolated neural and vascular cells, it was established that increased free fatty acids can trigger a toll-like receptor 4 (TLR4)-NFkB-dependent pro-inflammatory response in brain pericytes. This ultimately results in morphological changes in brain pericytes, associated with vascular dilation, breakdown of the extracellular matrix and increased vascular permeability [34]. This finding is compelling, as metabolic changes are thought to be a core component of neurodegenerative and neurodevelopmental disorders [110,111] and APOE, a key genetic risk factor for AD, plays a central role in lipid metabolism and transport [112,113]. However, whether metabolic changes induce defects in vascular cells during the onset of neurodegeneration in human remains to be determined.

Despite our ever-increasing appreciation of neural vasculature dysfunction in a range of neurological disorders, transcriptomic analysis of vascular phenotypes in neural pathologies is in its infancy. Future studies utilizing single cell technologies will provide invaluable insights into molecular mechanisms that drive vascular phenotypic changes associated with neural pathologies. Given that many of the vascular-associated neural pathologies are related to aging, the importance of environmental factors and epigenetic changes in mediating vascular breakdown will be an additional important line of study.

## 5. Outlook and Perspectives

### 5.1. Intercellular Communication

Inflammation is a process that involves multiple intercellular interactions and communication by means of secreted factors and chemokines on one side and receiving receptors on the other side. In order to address the heterocellular interactions within the context of complex tissues and inflammation, considerable effort has been spent on creating a comprehensive ligand-receptor database for humans [114]. Studies have utilized this Ramilowski database to uncover inter-cellular interactions in the developing mouse brain [19], heart [115], kidney [116] and tumors [117]. Furthermore, a number of bioinformatic tools [118,119,120] utilizing ligand-receptor databases such as the Ramilowski database [114] or REACTOME [121] are making intercellular communication analysis more accessible to single cell studies.

Bioinformatic tools have now also been employed to interrogate cellular interactions in the context of atherosclerosis [52]. In atherosclerosis, increased interactions between mesenchymal-like cells and immune cells have been reported in the *ApoE*^−/−^ mouse model [52]. Increased interacting ligand-receptor pairs were enriched in gene ontologies related to inflammation, leukocyte chemotaxis and ECM interactions, consistent with the important roles of these cellular processes in the context of atherosclerosis. In contrast, ligand-receptor interaction analysis of human heart vascular cells revealed that differences in cellular communication modules were dependent on vascular zonation, including specific patterning of *Notch* ligands. Arterial ECs mainly expressed *DLL4*, *JAG1* and *JAG2* that are recognized by *NOTCH2* in arterial SMCs, whereas venous EC expressed *DLL1* that is recognized by *NOTCH3* of venous SMCs [30]. Furthermore, neural vascular cells, microglia and neural cells of the developing mouse brain also display a complex intercellular communication network [19]. The authors reported that APOE, a protein implicated in Alzheimer’s disease and in atherosclerosis [122,123], is expressed by microglia during development and has reciprocal receptors in neural cells [19], suggesting APOE may play an important and currently undefined role during neural development.

In addition to the use of bioinformatic tools to identify potential cell–cell communication modules, a recently developed method, physically interacting cells (PIC-seq) attempts to directly probe inter-cellular interactions [124]. PIC-seq involves sorting single cells and physically interacting cell aggregates (PICs) into 384-well plates. Transcripts originating in single cells and PICs are computationally deconvoluted and putative ligand-receptor interactions are predicted. This method has been successfully applied to murine dendritic and T-cells both in vitro and in vivo and has the potential to further our knowledge of physically interacting cells in vascular inflammation models.

Together, these examples show the importance of cellular communication in homeostasis and pathogenesis of disease. Given that vascular cells show unique organ-dependent phenotypes and are involved in complex pathologies inflicting whole organs, future studies focusing on communication between different cell types will be critical for a better understanding of the contribution of vascular cells to disease.

### 5.2. Single Cell Epigenetics

Single cell technologies are rapidly developing. New adaptations of single cell technologies to epigenomic methods such as chromatin immunoprecipitation (ChIP)-seq [125,126], assay for transposase-accessible chromatin (ATAC)-seq [127,128] and chromosomal conformation capture for studying the 3D chromatin structure [129,130,131,132] are increasingly improving. Thus far, single cell ATAC-seq is the only widely employed single cell epigenomic technique [127,128]. This technique detects accessible open chromatin regions in the genome [133] and is available as a kit from 10x Genomics, making it a popular option. In addition, new elegant methods probing a combination of the epigenetic state and transcriptomics on a single cell level are being developed [134,135,136,137]. For instance, NOME-seq [138] is now being utilized to simultaneously probe DNA methylation and open chromatin in single cells [134,135]. Surprisingly, neither scATAC-seq, nor single cell methylation profiles have been generated for vascular cells. On the other hand, single cell ChIP-seq has already been successfully applied to vascular development. Mapping of H3K27ac in *Cdh5*-traced EC lineages showed unique profiles of active promoters and enhancers in the ECs isolated from 10 different tissues of E16.5 embryos [125]. This is consistent with scRNA-seq studies that identify organ-dependent EC heterogeneity [10], suggesting that unique epigenetic factors are likely to drive the identity of unique EC-subtypes. These epigenetic mechanisms remain to be identified.

Future utilization of the single cell epigenomic technologies in the context of vascular dysfunction and inflammation will help unravel the underlying chromatin changes that modulate transcription and drive pathological changes in cellular phenotype.

## 6. Concluding Remarks

Vascular diseases have been studied for decades, but we are only now starting to understand the full complexity and cellular heterogeneity underlying disease-associated pathophysiological conditions. A range of phenotypic switches and trans-differentiation of major vascular and immune cells have been reported with the help of single cell technologies (Table 2). It is now important to harvest the unprecedented amount of data and try to integrate them with the previously gathered knowledge on signaling pathways and cell interactions in the context of diseases such as atherosclerosis and neurodegenerative disorders. Intercellular communication studies are of particular interest as human diseases involve a multitude of cell types and the root cause(s) is often difficult to ascertain. Future studies focusing not only on transcriptomic changes, but also on underlying epigenomic alterations, will provide us with a more comprehensive understanding of cellular and molecular mechanisms that drive changes in vascular cells associated with a multitude of pathologies.

## Figures and Tables

**Table 1:** Cell type specific marker genes for vascular cell types from single cell data. Marker genes were derived from References [3,10,29]. Endothelial cells (ECs); smooth muscle cells (SMCs).

Cell Type	Marker Genes (scRNA-seq)
all ECs	*Kdr (Vegfr2)*, *Cdh5*, *Pecam1 (CD31)*, *Tie1*, *Flt1*, *Vwf*, *Icam2 (CD102)*
arterial ECs	*Hey1*, *Fbln5*, *Vegfc*, *Sema3g*, *Cytl1*, *Gkn3*, *Stmn2*, *Sox17*, *Bmx*, *Efnb2*
venous ECs	*Nr2f2*, *Lcn2*, *Vwf*, *Emcn*, *Scl38a5*, *Cfh*, *Apoe*
capillary ECs	*Mfsd2a*, *Rgcc*, *Ramp3*, *Cd300lg*, *Tgfb2 (A)*, *Glul (A)*, *Tfrc (V)*, *Car4 (V)*
lymphatic ECs	*Flt4*, *Prox1*, *Mmm1*, *Ccl21a*, *Mmrn1*, *Fgl2*, *Lyve1*, *Thy1*
all SMCs	*Myh11*, *Acta2*, *Tagln*, *Vim*, *Des*, *Myl9*, *Pdgfrb*, *Cspg4*, *Tcf21*
arterial SMCs	*Cnn1*
arteriole SMCs	*Acta2*, *Tagln*-high
venous SMCs	*Acta2*, *Tagln*-low
pericytes	*Pdgfrb*, *Cspg4*, *Des*, *Abcc9*, *Vtn*, *Anpep*, *Rgs5*
fibroblasts	*Dcn*, *Tcf21*, *Bgn*, *Eln*, *Col1a1*, *Col1a2*, *Pdgfra*

**Table 2:** Major single cell studies focusing on the vascular system.

Tissue, disease	Model	Main Finding	Reference
All vasculature	WT 8-week-old male C57BL6/J mice	Vascular cells show transcriptional heterogeneity that is organ-dependent and consistent with organ-specific specialization of vasculature.	[10]
Mouse aorta Atherosclerosis	12-week-old female C57/BL6 mice, 8 weeks of chow or Western diet	Detected three EC subpopulations in plaques and an increase in expression of contractile genes in ECs.	[47]
Mouse aorta Atherosclerosis	12-week-old male C57BL/6J WT and *ApoE*^−/−^ mice, chow diet	Detected activation of immune cells; inflammatory and progenitor-like state of non-immune cells; existence of SCA1^+^ SMC population	[52]
Mouse aorta Atherosclerosis	8- to 14-week-old male mice; *Myh11*-reporter, *Sca1*-reporter, *ApoE*^−/−^, cholesterol rich diet	Found increase in SCA1^+^ SMC population in atherosclerotic mice.	[48]
Mouse aorta Human aorta Atherosclerosis	*Myh11*-reporter, *Myh11*-driven *Tcf21* knockout (and control), *ApoE*^−/−^, high fat diet. Human: 3♂, 1♀, proximal-to-mid right coronary artery	Performed SMC lineage-tracing. Showed importance of TFC21 in humans and mice for the trans-differentiation of SMCs into fibroblasts (“fibromyocyte”).	[49]
Mouse aorta Atherosclerosis	6- to 8-week-old *Ldlr*^−/−^ C57BL/6J male mice, atherogenic diet; 8-week-old *ApoE*^−/−^ female mice, Western diet	Identified three subpopulations of macrophages in plaques: resident, inflammatory *Il1β*^hi^, *Trem2*^hi^.	[50]
Mouse aorta Atherosclerosis	*ApoE*^−/−^, *Ldlr*^−/−^, *Cx3cr1*-reporter, *LysM*-reporter C57BL6/J mice; D374Y-hPCSK9 transgenic mice, Western diet	Described heterogeneity of macrophages in plaques; Showed increased foamy macrophages as plaques increase in size.	[51]
Mouse aorta Atherosclerosis	8-week-old *Cx3cr1-*reporter mice, Western diet; recovery model – switch to a chow diet + injection of apolipoprotein B (*ApoB*) anti-sense oligonucleotide.	Performed tracing of *Cx3cr1*+ myeloid cells in plaque: *Cd11b*+ myeloid cells in the lesion; identified *Trem2*^hi^, *DNase1l3*^hi^, *Retnla*^hi^*Ear*^hi^, IFN signature^hi^, and “stem-cell like” macrophages.	[59]
Mouse heart MI	10- to 12-week-old male mice; myocardial infarction (MI) by permanent ligation of left anterior descending branch of the coronary artery; *Wt1*-reporter mice (epicardial tracing); *Tek*-reporter mice (endocardial tracing).	Post-MI: detected activation of fibroblasts, increase in myofibroblasts, occurrence of “matrifibrocyte” and increase in EC population.	[64]
Mouse heart MI	8 to 10-week-old *Pdgfb*-reporter mice; MI by permanent ligation of left anterior descending branch of the coronary artery.	Detected angiogenic, proliferative and pro-inflammatory EC subpopulations in border zone 7 days post-MI.	[67]
Human lung PAH	Human lung samples: healthy (*n* = 6) and idiopathic pulmonary arterial hypertension (*n* = 3).	Showed increase in EC angiogenesis and ECM production by SMCs and pericytes.	[74]
Mouse brain vasculature	10- to 19-week-old, *Cspg4*-reporter, *Pdgfrb*-reporter, *Pdgfra*-reporter, *Cldn5*-reporter, *Sm22*-reporter C57BL6/J mice.	Identified 1798 transcripts associated with EC zonation. Showed pericytes are not zonated, but segregate into 2 major clusters.	[3]
Developing mouse brain	E14.5 embryos, C57BL/6 background.	Identified 1710 unique ligand-receptor interactions between EC, pericytes, microglia and neural cells.	[19]
